# Supplementary material for: Embryonic keratin19+ progenitors generate multiple functionally distinct progeny to maintain epithelial diversity in the adult thymus medulla
Source: Nat Commun. 2023 Apr 12;14:2066. doi: 10.1038/s41467-023-37589-4 (PMC10097809; doi:10.1038/s41467-023-37589-4)
Supplement: Supplementary file 1 — Supplementary Information [file 41467_2023_37589_MOESM1_ESM.pdf]

Sup. Fig. 1

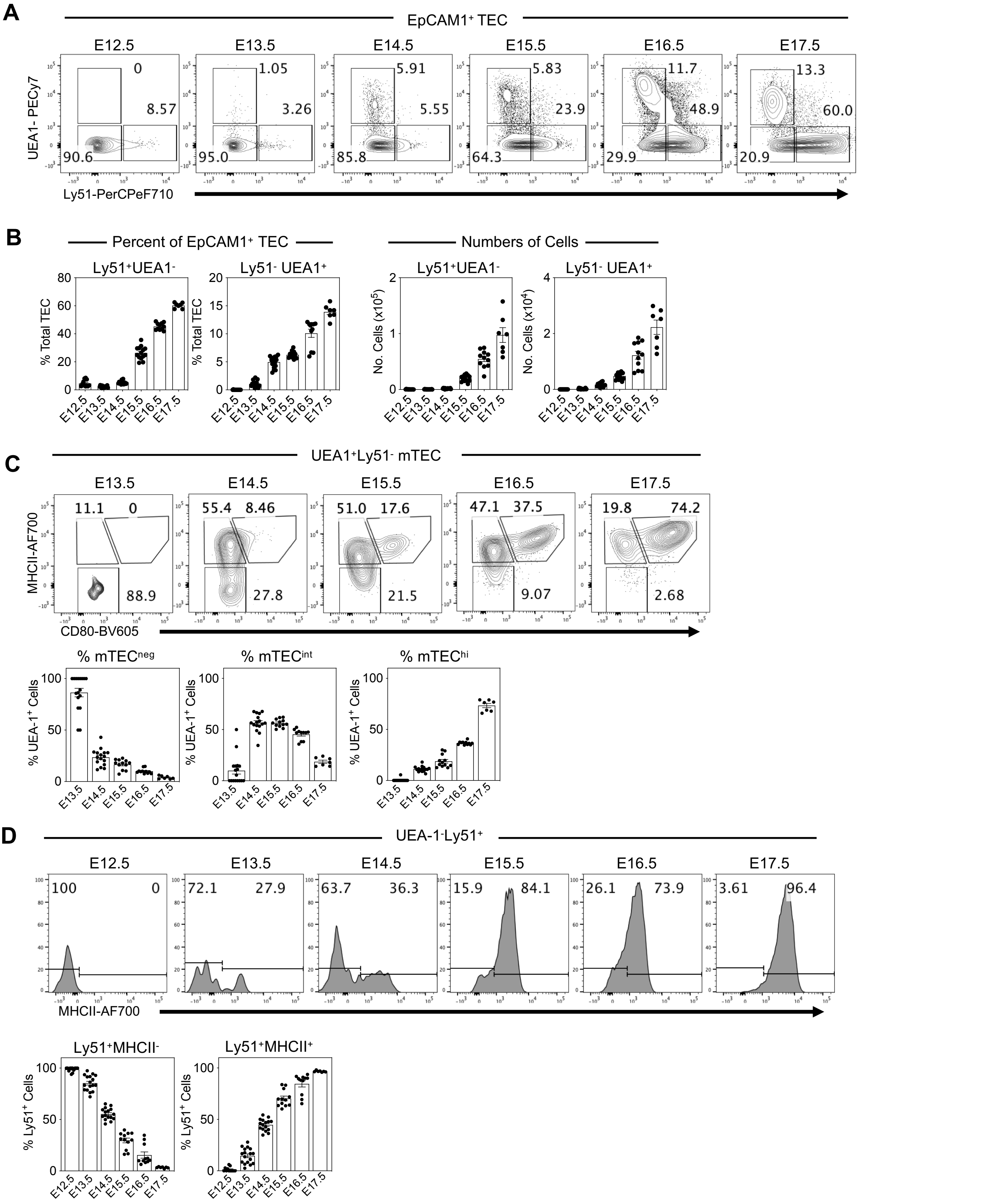

Supplementary Figure 1. Defining Stages in mTEC and cTEC Ontogeny.

A Representative FACS plots showing expression of UEA1 and Ly51 by EpCAM1$^+$ cells during ontogeny.

B Quantification of Ly51$^+$UEA1$^-$, and Ly51$^-$UEA1$^+$ subsets within EpCAM1$^+$ cells. **C** Representative FACS plots showing expression of MHCII and CD80 by UEA1$^+$Ly51$^-$ mTEC, to identify mTEC^{neg} (MHCII$^-$CD80$^-$), mTEC^{int} (MHCII^{int}CD80^{int}) and mTEC^{hi} (MHCII^{hi}CD80^{hi}), with corresponding quantitation E12.5 n=12, E13.5 n=17, E14.5 n=16, E15.5 n=12, E16.5 n=11, E17.5 n=7. **D** Representative FACS plots showing expression of MHCII by UEA1$^-$Ly51$^+$ TEC, with corresponding quantitation E12.5 n=12, E13.5 n=17, E14.5 n=16, E15.5 n=12, E16.5 n=11, E17.5 n=7. All data is from 3 independent experiments per gestational age. The data are shown as mean ± SEM.

Sup. Fig. 2

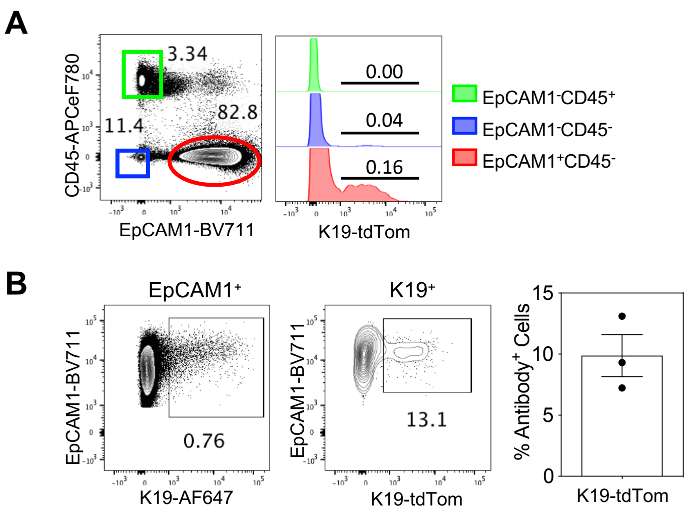

Supplementary Figure 2. Specificity and efficiency of *Krt19*^{ERTCre}
Fate mapping was induced in *Krt19*^{ERTCre}tdTom embryos at E15.5 by a single tamoxifen gavage, and embryonic thymi were analysed the following day, n=3. **A** K19-tdTom expression was determined in CD45^{+} haematopoietic cells (green), CD45^{-}EpCAM^{-}cells (blue) and EpCAM1^{+} TEC (red) by flow cytometry. **B** Representative FACS plots and quantitation showing expression of K19tdTom within K19-antibody^{+} EpCAM1^{+} cells. The data are shown as mean ± SEM.

Sup. Fig. 3

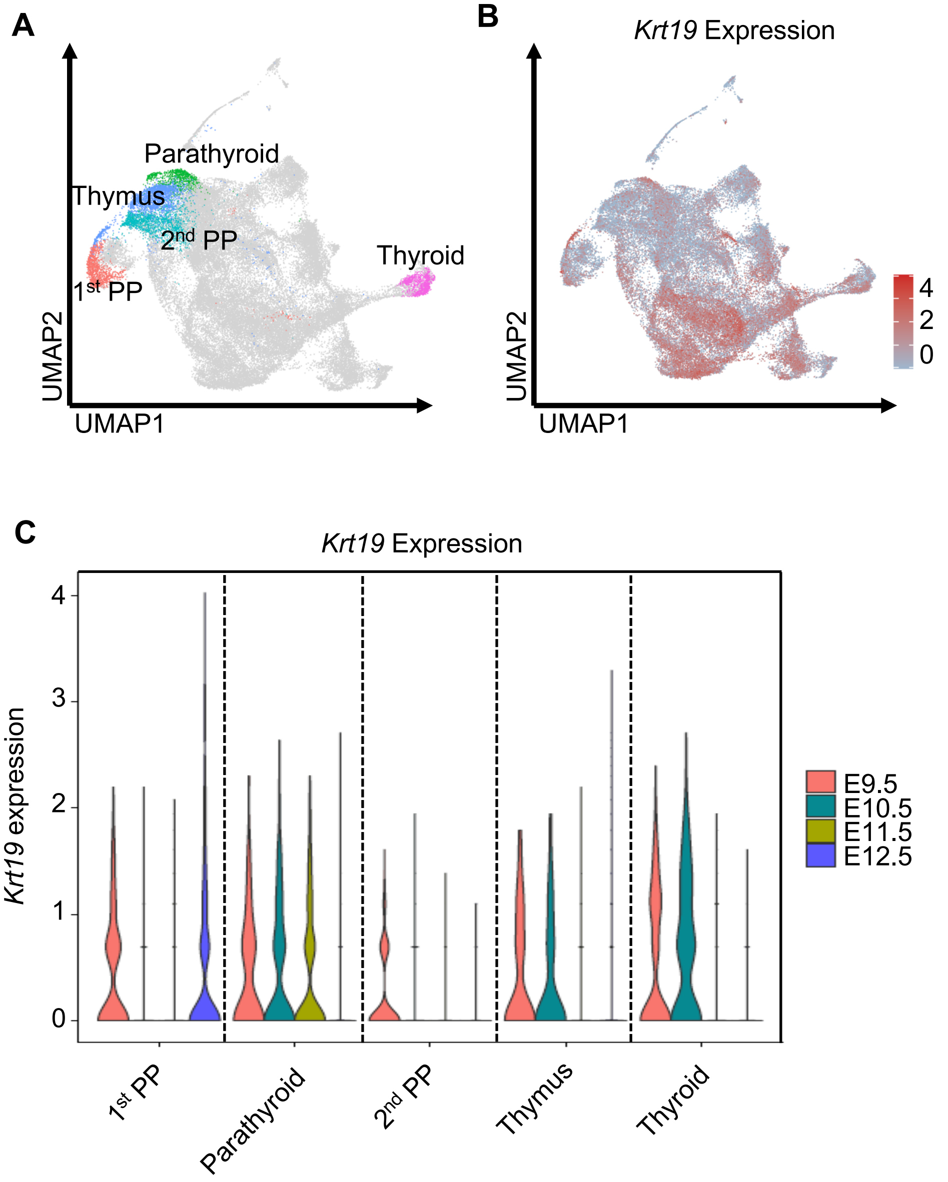

Supplementary Figure 3. *Krt19* expression prior to E12.5
A UMAP plot of single cell transcriptomic data of pharyngeal endoderm from E9.5-E12.5 showing clusters which have been annotated using published marker genes (ref - Magaletta et al. 2022). **B** UMAP plot showing *Krt19* expression across the pharyngeal endoderm. **C** Violin plots showing *Krt19* expression within different cell types within the pharyngeal endoderm between E9.5 and E12.5.

Sup. Fig. 4

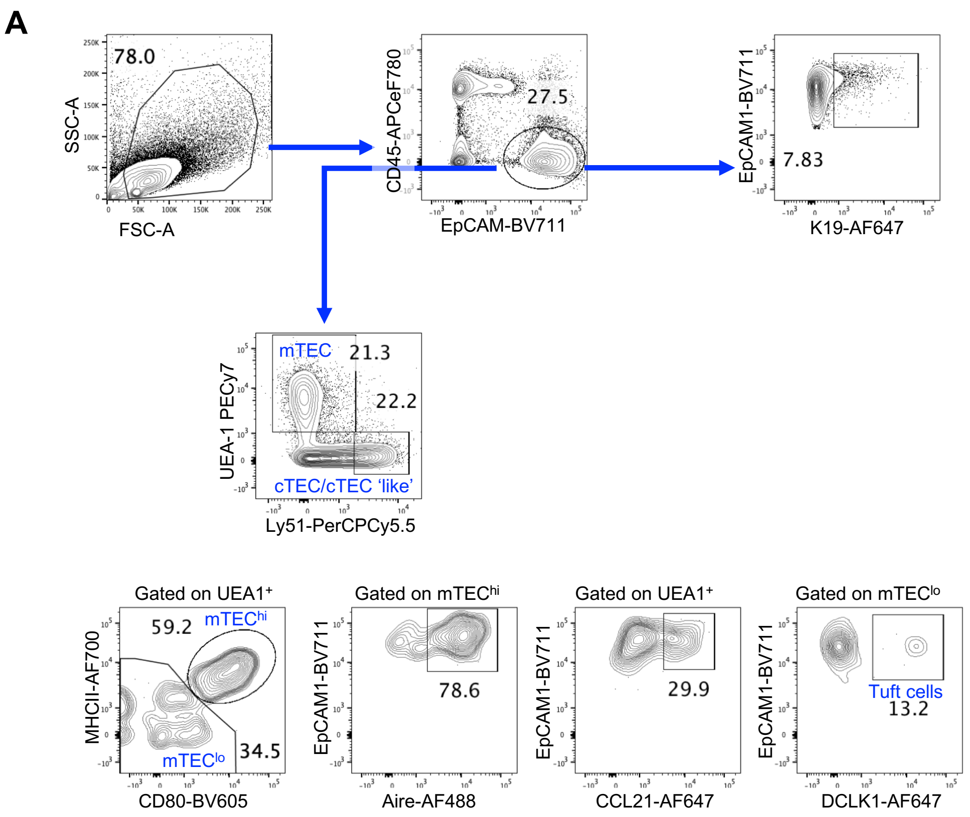

Supplementary Figure 4. Gating strategies used for flow cytometry
A Gating for live cells based on FSC/SSC, and identification of EpCAM1⁺ thymic epithelial cells (TEC), K19⁺ TEC, UEA1⁺ mTEC, Ly51⁺ cTEC/cTEC 'like', mTEC^{lo}, mTEC^{hi}, Aire⁺ mTEC^{hi}, CCL21⁺ mTEC, and thymic tuft cells. Gating strategy used in Fig1, 3-9.
