# Supplementary material for: Embryonic keratin19+ progenitors generate multiple functionally distinct progeny to maintain epithelial diversity in the adult thymus medulla
Source: Nat Commun. 2023 Apr 12;14:2066. doi: 10.1038/s41467-023-37589-4 (PMC10097809; doi:10.1038/s41467-023-37589-4)
Supplement: Supplementary file 3 — Reporting Summary [file 41467_2023_37589_MOESM3_ESM.pdf]

nature portfolio

Corresponding author(s): Graham Anderson

Last updated by author(s): Mar 13, 2023

Reporting Summary

Nature Portfolio wishes to improve the reproducibility of the work that we publish. This form provides structure for consistency and transparency in reporting. For further information on Nature Portfolio policies, see our Editorial Policies and the Editorial Policy Checklist.

Statistics

For all statistical analyses, confirm that the following items are present in the figure legend, table legend, main text, or Methods section.

n/a	Confirmed	
☐	☒	The exact sample size (*n*) for each experimental group/condition, given as a discrete number and unit of measurement
☐	☒	A statement on whether measurements were taken from distinct samples or whether the same sample was measured repeatedly
☐	☒	The statistical test(s) used AND whether they are one- or two-sided<br>*Only common tests should be described solely by name; describe more complex techniques in the Methods section.*
☐	☒	A description of all covariates tested
☒	☐	A description of any assumptions or corrections, such as tests of normality and adjustment for multiple comparisons
☐	☒	A full description of the statistical parameters including central tendency (e.g. means) or other basic estimates (e.g. regression coefficient) AND variation (e.g. standard deviation) or associated estimates of uncertainty (e.g. confidence intervals)
☐	☒	For null hypothesis testing, the test statistic (e.g. *F*, *t*, *r*) with confidence intervals, effect sizes, degrees of freedom and *P* value noted<br>*Give P values as exact values whenever suitable.*
☒	☐	For Bayesian analysis, information on the choice of priors and Markov chain Monte Carlo settings
☒	☐	For hierarchical and complex designs, identification of the appropriate level for tests and full reporting of outcomes
☒	☐	Estimates of effect sizes (e.g. Cohen's *d*, Pearson's *r*), indicating how they were calculated

Our web collection on statistics for biologists contains articles on many of the points above.

Software and code

Policy information about availability of computer code

Data collection	Not applicable
Data analysis	Not applicable

For manuscripts utilizing custom algorithms or software that are central to the research but not yet described in published literature, software must be made available to editors and reviewers. We strongly encourage code deposition in a community repository (e.g. GitHub). See the Nature Portfolio guidelines for submitting code & software for further information.

Data

Policy information about availability of data

All manuscripts must include a data availability statement. This statement should provide the following information, where applicable:

- Accession codes, unique identifiers, or web links for publicly available datasets
- A description of any restrictions on data availability
- For clinical datasets or third party data, please ensure that the statement adheres to our policy

All data relevant to this manuscript is contained within the submitted figures

Human research participants

Policy information about studies involving human research participants and Sex and Gender in Research.

Reporting on sex and gender	*Use the terms sex (biological attribute) and gender (shaped by social and cultural circumstances) carefully in order to avoid confusing both terms. Indicate if findings apply to only one sex or gender; describe whether sex and gender were considered in study design whether sex and/or gender was determined based on self-reporting or assigned and methods used. Provide in the source data disaggregated sex and gender data where this information has been collected, and consent has been obtained for sharing of individual-level data; provide overall numbers in this Reporting Summary. Please state if this information has not been collected. Report sex- and gender-based analyses where performed, justify reasons for lack of sex- and gender-based analysis.*
Population characteristics	*Describe the covariate-relevant population characteristics of the human research participants (e.g. age, genotypic information, past and current diagnosis and treatment categories). If you filled out the behavioural & social sciences study design questions and have nothing to add here, write "See above."*
Recruitment	*Describe how participants were recruited. Outline any potential self-selection bias or other biases that may be present and how these are likely to impact results.*
Ethics oversight	*Identify the organization(s) that approved the study protocol.*

Note that full information on the approval of the study protocol must also be provided in the manuscript.

Field-specific reporting

Please select the one below that is the best fit for your research. If you are not sure, read the appropriate sections before making your selection.

- [x] Life sciences
- [ ] Behavioural & social sciences
- [ ] Ecological, evolutionary & environmental sciences

For a reference copy of the document with all sections, see nature.com/documents/nr-reporting-summary-flat.pdf

Life sciences study design

All studies must disclose on these points even when the disclosure is negative.

Sample size	Sample size for each of our experiments is shown in the relevant figure legends. This was chosen based on previous experience with similar experiments, and in order to reproducibly detect specific effects. Statistical methods were not used to predetermine sample size.
Data exclusions	No data was excluded from analysis.
Replication	All experiments were repeated so data in the manuscript are from at least three or four independent experiments that gave similar results. The figure legends contain exact details of the numbers of repeats for each experiment.
Randomization	No randomization of mice was performed.
Blinding	Investigators were not blinded to mouse genotypes in our experiments. This was to help ensure that appropriate sample size was achieved by sacrificing the minimum number of mice required to achieve consistent data.

Reporting for specific materials, systems and methods

We require information from authors about some types of materials, experimental systems and methods used in many studies. Here, indicate whether each material, system or method listed is relevant to your study. If you are not sure if a list item applies to your research, read the appropriate section before selecting a response.

Materials & experimental systems

n/a	Involved in the study	
[ ]	[x]	Antibodies
[x]	[ ]	Eukaryotic cell lines
[x]	[ ]	Palaeontology and archaeology
[ ]	[x]	Animals and other organisms
[x]	[ ]	Clinical data
[x]	[ ]	Dual use research of concern

Methods

n/a	Involved in the study	
[x]	[ ]	ChIP-seq
[ ]	[x]	Flow cytometry
[x]	[ ]	MRI-based neuroimaging

Antibodies

Antibodies used	For flow cytometry, the following antibodies were used: CD45-APC eFluor780 (A20, eBioscience, Cat no: 47-0451-82, Lot no: 2375407), EpCAM-BV711 (G8.8 Biolegend, Cat no: 118233, Lot no: B339822), UEA-1 biotin (Vector labs, Cat no: B-1065, Lot no: ZF1204), Ly51 PerCPeF710 (BP-1, eBioscience, Cat no: 46-5891-82, Lot no: 2134423), MHCII AF700 (M5/114.15.2, eBioscience, Cat no: 56-5321-82, Lot no: 2210930), CD80-BV605 (16-10A1, Biolegend, Cat no: 104729, Lot no: B340200), SSEA-1 BV421 (MC-480, Biolegend, Cat no: 125614, Lot not: B311195) Biotinylated antibodies were detected using Streptadvidin-PECy7 (eBioscience, Cat no: 25-4317-82, Lot no: 2034750). Intracellular staining was performed following fixation with 5% formalin solution (Sigma Aldrich) and antibodies used were: Aire AF488 (5H12, Cat no: 53-5934-82, Lot no: 2312434), K19 (EP1580Y, Abcam, Cat no: ab52625, Lot no: GR3384962-1), DCLK1 (DCAMKL1, Abcam, Cat No: ab31704, Lot no: GR3357375-3), CCL21 (LifeSpan Technologies, Cat no: LS-C104634, Lot no: 55059), Relb (Santa Cruz, C-19, Cat no: sc-226, Lot no: F1912). Unconjugated intracellular antibodies were detected using chicken anti-rabbit AF647 (Invitrogen, Cat no: A21443, Lot no: 1881092), or donkey anti-rabbit AF488 (Invitrogen, Cat no: A21206, Lot no: 2072687).<br><br>For Immunofluorescence the following antibodies were used: ERTR5 (van Vliet 1985); detected using goat anti-rat IgM AF647 (Invitrogen, Cat no: A21248, Lot no: 2160421), and K19 (EP1580Y, Abcam, Cat no: ab52625, Lot no: GR3384962-1); detected using donkey anti-rabbit IgG AF555 (Invitrogen, Cat no: A31572, Lot no: 2017396). Retention of K19-tdTom in fate mapping experiments was achieved by fixing thymus tissue in 2% PFA for 2 hours, followed by 20% sucrose overnight, before being snap frozen. Subsequent cryosections were stained with the following antibodies: K5 AF647 (Abcam, EP1601Y Cat no: ab193895, Lot no: GR3416059-2), Aire AF488 (5H12, Cat no: 53-5934-82, Lot no: 2312434), EpCAM biotin (G8.8, Biolgend, Cat no: 118204, Lot no: B273843); detected using Streptavidin AF647 (Invitrogen, Cat no: S21374, Lot no: 1990312), DCLK1 (DCAMKL1, Abcam, Cat No: ab31704, Lot no: GR3357375-3); detected using donkey anti-rabbit IgG AF555 (Invitrogen, Cat no: A31572, Lot no: 2017396).
Validation	All antibodies were optimised for flow cytometry and confocal microscopy by titration prior to use in all experiments.

Animals and other research organisms

Policy information about studies involving animals; ARRIVE guidelines recommended for reporting animal research, and Sex and Gender in Research

Laboratory animals	Species: Mus Musculus. All mice were used at age 8-12 weeks unless stated otherwise. Strains used:
Wild animals	Not Applicable
Reporting on sex	A mixture of male and female mice were analysed in this study. The data from each sex has been pooled for analysis and cannot be broken down.
Field-collected samples	Not Applicable
Ethics oversight	All mouse studies were performed in accordance with local and home office guidance

Note that full information on the approval of the study protocol must also be provided in the manuscript.

Flow Cytometry

Plots

Confirm that:

- [x] The axis labels state the marker and fluorochrome used (e.g. CD4-FITC).
- [x] The axis scales are clearly visible. Include numbers along axes only for bottom left plot of group (a 'group' is an analysis of identical markers).
- [x] All plots are contour plots with outliers or pseudocolor plots.
- [x] A numerical value for number of cells or percentage (with statistics) is provided.

Methodology

Sample preparation	All samples were prepared from freshly isolated mouse organs by either mechanical disruption or enzymatic digestion.
Instrument	Becton Dickinson Fortessa Flow Cytometer
Software	FACS DIVA software (BD Biosciences) and FlowJo software (TreeStar)
Cell population abundance	Flow cytometry cell sorting was not used in this study
Gating strategy	Viable cells were identified by flow cytometry using FSC/SSC gating. Discrimination of cells staining postively or negatively for antibodies was achieved by gating on samples stained with either isotype controls, or following omission of the primary antibody.

- [x] Tick this box to confirm that a figure exemplifying the gating strategy is provided in the Supplementary Information.
